# Tuberculous Disease Affecting the Tarsus

**Published:** 1908-02-15

**Authors:** 


### Points in Surgery.

### TUBERCULOUS DISEASE AFFECTING THE TARSUS.

Very few surgical conditions are more disappointing in their results than is tuberculosis of the tarsus, at any rate, as it occurs in the hospital patient who is necessarily unable to afford the expense of treatment by prolonged rest in a suitable neighbourhood. It must be remembered that tuberculosis, whether it be medical or surgical, is an expensive disease to arrest or to cure, if, indeed, it can ever be called cured. Judging by one's personal experience, it would seem that the only term which can justifiably be applied to this affection when it has once given rise to obvious clinical manifestations is "quiescent," however great the improvement under proper treatment may appear to be. The phrase "when it has once given rise to obvious clinical manifestations" is designedly used, because pathologists tell us that considerably over fifty per cent. of all bodies examined post-mortem exhibit miscroscopical evidence of old tuberculous foci, although the patient's attention was never called to their existence by any symptom, and although he may have died of some quite different disorder. To explain this we must suppose that the possessor of such a focus was exposed to infection with the tubercle bacillus, but that his inherent resistance to the micro-organism was so great that it was never really able to exert its malign power upon him.

Unfortunately this is rarely the case when tuberculosis attacks the tarsus. On the contrary, apart from the lung and lymphatic glands, there is no tissue in the body which is attacked by the tubercle bacillus with such readiness as cancellous bone. As in most infective diseases of bone, the infection is probably a hæmic one: that is to say, that the bacillus is an inhabitant of the blood stream (and it is probable that all of us possess in our vascular system some tubercle bacilli). As long as the resistance of the tissues is normal the bacillus is inert. But let that resistance be momentarily lowered in any part, and what is the result? Immediately the bacilli swoop down upon the damaged tissue, if one may use the phrase, and exert their influence. In the case of tuberculosis there can be little reason to doubt that the predisposing cause which leads to this result is nearly always an injury. If a careful history is extracted from patients suffering from surgical tuberculosis, especially that of bones and joints, it will be found that there has been some antecedent injury in the great majority of cases. It is true that this has often been so slight as almost to have passed unnoticed, or to have been dismissed without further thought as a sprain, only to be recalled later, when the injured part becomes the seat of constant pain. In other words, disease is, pathologically speaking, the expression of some loss of equilibrium between the vitality of the tissues and the power of infectivity possessed by micro-organisms which are normal inhabitants of the blood-stream.

The first indication of tuberculosis of the tarsus is generally a continuous pain constantly referred to one part. It may be aggravated by flexing the foot, as in the act of walking. In other cases this may be absent, but there is a localised tender spot, which is exceedingly painful if it is knocked or subjected to pressure. Such spots are situated over the inner margin of the foot, along the first metatarsal, internal cuneiform and scaphoid bones, and on the outer side over the cuboid, or in the heel if the calcaneum is affected. It is useful to remember that such a tender spot, which is excessively painful, even on light percussion, and causes the patient to shrink away, is highly suggestive of disease of the underlying bone or its periosteum. Later the affected portion becomes swollen, shiny, and pulpy; and this is an indication that the periosteum of the bone is now involved in the process. Unlike acute inflammatory swellings, which are bright red, this is blueish purple, a colour which is so characteristic of tuberculous disease that the nature of the process may be suspected almost at first sight. The clinical course of the disease varies slightly, according to the position of the bone first attacked. If it be in the anterior part of the foot the swelling may arrive at a considerable size before there is any limitation of movement at the ankle-joint; but if the disease starts posteriorly this joint is involved early, especially if the primary focus is in the astragalus. In this case all movements at the joint are soon limited, and may even be absent, and the foot is held in the equinus position. But in a case in which the disease is not arrested early, the clinical course only varies slightly in any case because the process spreads by slow but sure degrees to adjacent joints and bones, so that finally nearly all the tarsus, as well as the ankle-joint, is involved. Finally suppuration occurs, an abscess forms, and breaks through the skin at some point, leaving a sinus which continues to discharge tuberculous *débris*, and is a source of danger to the patient on account of the risk of septic infection. Such is the picture of an extreme case. It must not be supposed that the clinical course is invariably as bad as this, but the prognosis is never good. If, however, the disease is diagnosed early and treatment effectively carried out, the disease may be arrested. The foot should be kept absolutely at rest, in a plaster of Paris splint if necessary, and constitutional anti-tuberculous treatment should be adopted. If possible the patient should not be allowed to walk on the foot until at least three months has elapsed after his pain has quite disappeared. If, in spite of this treatment, a localised abscess forms, it may be opened and scraped, and allowed to granulate up, and rest again tried. But if the disease still gains ground, it is wise to do a Syme's amputation while it is yet possible, for when the skin over the os calcis is involved, an amputation will have to be performed in the lower third of the leg. This is not nearly so satisfactory as a Syme's amputation, which gets rid of all the disease, and leaves an excellent stump to which an artificial foot can be easily adjusted.

